# Horizontal gene transfer between *Wolbachia *and the mosquito *Aedes aegypti*

**DOI:** 10.1186/1471-2164-10-33

**Published:** 2009-01-20

**Authors:** Lisa Klasson, Zakaria Kambris, Peter E Cook, Thomas Walker, Steven P Sinkins

**Affiliations:** 1 Department of Zoology, University of Oxford, South Parks Road, Oxford, OX1 3PS, UK

## Abstract

**Background:**

The evolutionary importance of horizontal gene transfer (HGT) from *Wolbachia *endosymbiotic bacteria to their eukaryotic hosts is a topic of considerable interest and debate. Recent transfers of genome fragments from *Wolbachia *into insect chromosomes have been reported, but it has been argued that these fragments may be on an evolutionary trajectory to degradation and loss.

**Results:**

We have discovered a case of HGT, involving two adjacent genes, between the genomes of *Wolbachia *and the currently *Wolbachia*-uninfected mosquito *Aedes aegypti*, an important human disease vector. The lower level of sequence identity between *Wolbachia *and insect, the transcription of all the genes involved, and the fact that we have identified homologs of the two genes in another *Aedes *species (*Ae. mascarensis*), suggest that these genes are being expressed after an extended evolutionary period since horizontal transfer, and therefore that the transfer has functional significance. The association of these genes with *Wolbachia *prophage regions also provides a mechanism for the transfer.

**Conclusion:**

The data support the argument that HGT between *Wolbachia *endosymbiotic bacteria and their hosts has produced evolutionary innovation.

## Background

*Wolbachia pipientis *is an intracellular inherited bacterium found in arthropods, where it manipulates host reproduction using phenotypes such as cytoplasmic incompatibility (CI), male killing, parthenogenesis and feminization, and can spread rapidly through insect populations [1]. It is also an obligate mutualist of a number of filarial nematode species [2].

Several cases where sections of the *Wolbachia *genome, sometimes large, have been transferred to the host chromosomes are now known in both insects and nematodes [3-5]. These are either recent events where *Wolbachia *and host sequences are highly similar or involve extensive pseudogenization [4]. Transcription was reported for 2% of the genes transferred to *Drosophila ananassae *but the levels were estimated to be 10^4 ^to 10^7 ^fold lower than for a control gene, *act5C *[5,6], and it has been argued that this could represent background transcriptional noise (as occurs for many pseudogenes) rather than functional expression [7,8] – translation has yet to be demonstrated. It has therefore been suggested that these fragments are on an evolutionary trajectory to degradation by neutral mutation and play no significant part in host evolution [8]. If so they would be analogous to the non-functional nuclear fragments of mitochondrial DNA present in some animal genomes [9], which are transient and in the process of decay.

The case has therefore been made that if *Wolbachia*-insect HGT has evolutionary significance, both longevity and integration into host biology would need to be demonstrated [8]; and furthermore that we would expect to see *Wolbachia*-like genes in species that do not currently harbour *Wolbachia *but presumably did in the past. Both phylogenetic analyses and theory suggest that *Wolbachia *can be lost over time from host species by a variety of mechanisms [10-12]. *Aedes aegypti*, the most important mosquito vector of human dengue fever and various other arboviruses, is naturally *Wolbachia*-uninfected but has been shown to be able to support *Wolbachia *following artificial transinfection – with both high rates of maternal inheritance and the expression of high levels of CI [13]. The examination of its sequenced genome [14] for any genes that could have originated in *Wolbachia *was therefore undertaken.

## Results and discussion

**Figure 1:**
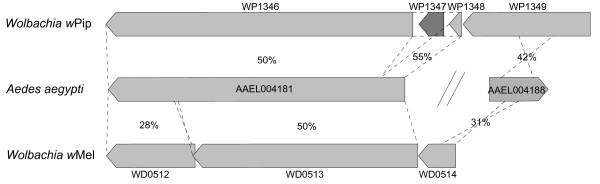
**A. Percent amino acid identities shared between *Aedes aegypti *and *Wolbachia *genes/gene regions**.

We have discovered a case of HGT involving adjacent genes in the genomes of *Ae. aegypti *and two *Wolbachia *strains. The *Ae. aegypti *gene AAEL004181 shares around 50% amino acid identity with two genes in the genome of *Wolbachia *strain *w*Pip [15] from the mosquito *Culex quinquefasciatus*, WP1348 and WP1346), which were probably originally a single gene split by insertion of IS element WP1347, and also with WD0513 in strain *w*Mel from *Drosophila melanogaster *[16]. The adjacent *Ae. aegypti *gene AAEL004188 shows partial similarity to *w*Pip WP1349 and to *w*Mel WD0514 and is inverted compared to the *Wolbachia *genes. The intergenic region between AAEL004181 and AAEL004188 is around 15 Kb (Figure 1). The level of sequence identity and the fact that adjacent sets of genes are involved provide a robust case for an HGT event.

**Figure 2:**
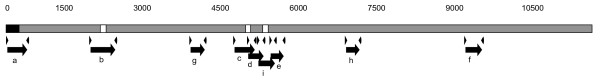
**Map showing positions of oligonucleotide PCR primers for gene AAEL004181 and the positions of introns (white boxes) present in the *Aedes aegypti *Vectorbase annotation for this gene (see Table 1 for primer sequences, product sizes and conditions used in the study)**. The black box represents an extension of the gene compared to the annotation in Vectorbase. Primers AAEL004181b, c and i were designed to span these introns produced RT-PCR products identical in size to those produced by genomic PCR, confirming that the introns were in fact mis-annotations; RT-PCR using AAEL004181a extended the 5' end of the exon.

RT-PCR analysis was conducted and confirmed that *Ae. aegypti *genes AAEL004181 and AAEL004188 were transcribed in both male and female adult mosquitoes. The *Wolbachia w*Pip genes WP1348 and WP1346 were clearly amplified by RT-PCR in adult *Cx. quinquefasciatus *of both sexes, indicating that they are also transcribed. Primers AAEL004181 b, c and i were designed to span three introns present in the *Aedes aegypti *Vectorbase annotation for AAEL004181; based on product size from RT-PCR amplification (Figure 2 and Table 1), all of these three introns proved to be mis-annotations and it was concluded that no introns are present. The apparent absence of introns, which would be particularly unusual for a mosquito gene of the size of AAEL004181, is suggestive of a bacterial origin.

**Table 1:** Sequences (5'-3'), optimal annealing temperatures (°C) and amplified fragment sizes (base pairs) for primers used in the study.

	primer sequence	product size (bp)	optimal annealing temperature (°C)
WP1346a*	F-TGGTTGGTCACTACCACGAA R-ACCATTCGGCACTGAAACAT	432	54

WP1346b	F-TGAGCATGGTCGTTTATTGG R-CGCACATCTTTCATCCAGAG	453	54

WP1346c	F-TCAATCTCGCAAGTTGATGC R-AAATGACCTTGAACGGAAGC	529	54

WP1348*	F-ACGACAAGCCTTTTCCTTTG R-TAATATTGCCGGGCTTGTTC	355	54

WP1349a*	F-TGGATGTGCGACGTTCTAAG R-TCGGCTGGTAATCCTTTTTG	399	54

WP1349b	F-GTGGAATTTTGAAGGCCAAG R-AGGCCCAACATTTTCTTGTG	372	54

AAEL004181a*	F-TTCTCCGACCAGATTTTTCC R-AGAAATGTCCCGCCCTTATC	370	54

AAEL004181b*^^†^	F-GAACAAGGGGATCAAGCAAA R-CTTGAATGACCCGAGTGAGA	475	54

AAEL004181c*^	F-CGGAACTCTGGTGGGTACAT R-AGACGTTCGCTTGAAAATCG	388	55

AAEL004181d	F-catcggttattgaaccggatac R-caacttcactattctgccaacg	296	54

AAEL004181e	F-tcttccgataggttacggattg R-tcgatgtataagcctccatcaac	251	54

AAEL004181f*^†^	F-ACAACCAGTGGAATCCTTCG R-GTTCTCATTTGCGACCCAAT	318	55

AAEL004181g^†^	F-cgctgaaactgtacagacaagg R-tttcattcgtgttgaagtggtc	273	54

AAEL004181h^†^	F-acaccactttcgattgtcattg R-ggcccttcatagctgtagtgac	260	54

AAEL004181i*^	F-GCCATCATCAGGAACCAATC R-CTGATTGCAGCGAGAAATGA	317	55

AAEL004188*^†^	F-TGGACACAAAGACCCATTCA R-AAAACTGGGTGCTTCCATTG	447	53

*denotes primers used for RT-PCR to determine gene expression. ^denotes primers designed to span three introns present in the *Aedes aegypti *Vectorbase annotation for AAEL004181. †denotes primers used for sequencing *Aedes mascarensis *PCR products.

**Figure 3:**
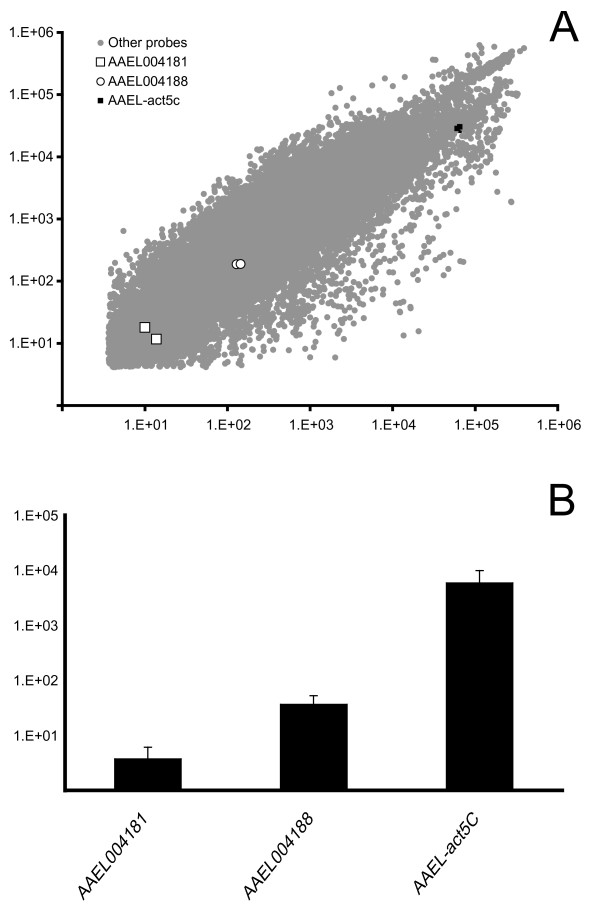
**A. Transcript levels of *Ae. aegypti *genes, AAEL004181 and AAEL004188, relative to whole genome transcription**. Microarrays incorporating two distinct 60-mer probes for each annotated *Ae. aegypti *gene were used to investigate transcript abundance in pools of adult female mosquitoes. In the example shown, array 1 was hybridised with cRNA from young mosquitoes (0–2 days post-eclosion) and Array 2 with cRNA from older mosquitoes (14–16 days post-eclosion). Log cyanine-3 signal intensity is shown for all probes (*n *= 29 840) significantly above background fluorescence (see methods). B. AAEL004181, AAEL004188 and *act5C *genes expression in *Aedes aegypti *females as monitored by quantitative RT-PCR. The values shown are the average of three different assays on independent samples. Error bars indicate standard error.

Full genome microarrays for *Aedes aegypti *were hybridized to cDNA from adult females, as shown in Figure 3. All probes with hybridization signal levels significantly above background (see methods) were ranked in order of signal intensity; 4.9% of these probes (or approximately 15% of all probes) showed lower signal intensity than was seen for either of the two AAEL004181 probes, while 28.2% of probes significantly above background (42% of all probes) showed lower signal intensity than was seen for either of the two AAEL004188 probes. Quantitative RT-PCR data for AAEL004188, AAEL004188 and he *act5C *gene used as a control by Hotopp *et al*. [5] matched the array results, as shown in figure 3. The *act5C *gene is, as the authors note, highly and constitutively expressed and may in fact be an overly stringent point of comparison to assess whether horizontally transferred genes are likely to be functional. In the microarray data a number of genes of known function showed very similar or lower hybridization intensities relative to the two genes of interest; for example the probes for AAEL012836 (*Cytochrome B561*) and AAEL002230 (encoding a chromatin helicase DNA binding protein) showed very similar hybridization intensities as AAEL004181, while AAEL013002 (*Cdk9*) and AAEL010226 (*Daughterless*) showed very similar hybridization levels as AAEL004188. Based on these data it is considered likely that both AAEL004181 and AAEL004188 are expressed, functional genes, although obviously definitive proof of this will require the raising of antibodies followed by protein studies.

Close homologs of the two *Ae. aegypti *genes could not be found in other sequenced mosquito/insect genomes such as *Anopheles gambiae *[17]. Various PCR primer pairs designed for the *Aedes aegypti *genes AAEL004181 and AAEL004188 failed to amplify PCR products from several other fellow subgenus *Stegomyia *members, but did amplify products for both genes from *Ae. mascarensis *(Table 2). This species, from Mauritius, is able to produce sterile offspring in laboratory crosses with *Ae. aegypti *[18]. *Ae. mascarensis *PCR products from AAEL004181 primers b & f plus g & h (see Figure 2 and Table 1), located in diverse regions of the gene, were sequenced and shared a mean 97% nucleotide identity over 1278 base pairs with *Ae. aegypti *AAEL004181. Diverged homologs of the two genes may well be present in other more distant *Aedes *species, but could not be detected here. Thus, if the direction of the HGT was from *Wolbachia *to host it would have occurred at least prior to the species divergence of *Ae. aegypti *and *Ae. mascarensis *and indeed the accumulation of the 15 Kb of non-coding DNA between the two genes would likely have required a considerable period of time (although it is not possible to make any precise time estimates from the data available).

**Table 2:** PCR amplification results from genomic DNA to examine the distribution of *Ae. aegypti *genes AAEL004181 and AAEL004188, plus presence/absence of *Wolbachia*, among other species in the *Aedes *subgenus *Stegomyia*.

	***aegypti***	***albopictus***	***simpsoni, heischi, calceatus, metallicus, soleatus***	***mascarensis***
***wsp *– *Wolbachia***	-	+	-	-
**Aeg S7 – control**	+	+	+	+
**AAEL004181a**	+	-	-	+
**AAEL004181b**	+	-	-	+
**AAEL004181c**	+	-	-	+
**AAEL00418d**	+	-	-	-
**AAEL004181e**	+	-	-	-
**AAEL004181f**	+	-	-	+
**AAEL004188**	+	-	-	+

+ indicates a clear PCR product of the correct size.

The *w*Pip genes are located at the end of a genomic prophage region, providing a putative mechanism for the HGT. *Wolbachia *have been shown to contain phage particles by EM in several studies; WO prophage have been shown to be highly variable and rapidly evolving regions in the genomes of mosquito *Wolbachia*, and non-congruent with host phylogeny [19-24], strongly suggesting that lateral transfer of phage between *Wolbachia *strains has occurred. The two *w*Mel genes WD0512 and WD0513 are part of an operon that also contains the ankyrin repeat domain (ANK) encoding gene WD0514. This operon is present in mod+ strain variants of *w*Mel (able to induce CI in males) but not in the related mod- strain *w*Au (unable to induce CI) [25]. The operon is located in a region of the *w*Mel genome that was shown to be missing in *w*Au, WD0506-WD0518 in *w*Mel, and in fact all these genes have homologs in the prophage regions of the *w*Pip genome, except for the ANK gene WD0514. Therefore, although not annotated as prophage [16], these genes in *w*Mel are likely to be remnants of an old prophage region, the rest of which has been deleted or rearranged.

The *Ae. aegypti *gene AAEL004181 also shares considerably lower amino acid similarity with a group of genes in the *Ae. aegypti*, *Anopheles gambiae *and *Culex pipiens *genomes. One of these *Ae. aegypti *genes showed female salivary gland specific expression and was named aaSGS1 (SGS = Salivary Gland Specific). This gene and homologs in *Anopheles *are candidate *Plasmodium *sporozoite receptors [26,27]. It has already been suggested that the SGS-type mosquito genes might have arisen from an ancient transfer between *Wolbachia *and mosquitoes, but with weak support [26,27].

**Figure 4:**
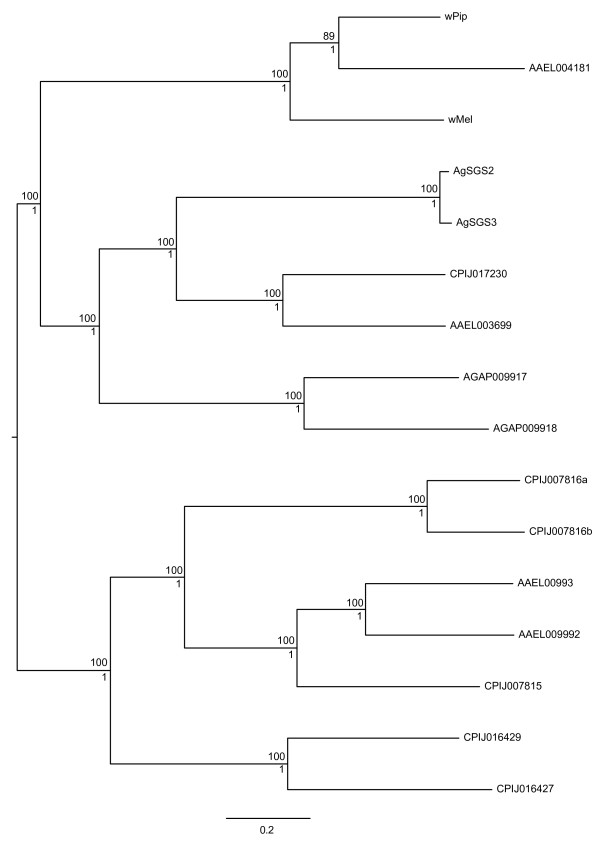
**Phylogenetic tree of the SGS genes, AAEL004181 in *Ae. aegypti *and homologous sequences in *Wolbachia***. The fragments of the AAEL004181 homolog sequenced from *Ae. mascarensis *are highly similar to AAEL004181, and are not shown here. Boostrap values from the ML analysis of 1000 replicates are shown above the branches and posterior probabilities from the Bayesian analysis are shown beneath the branches.

An alternative hypothesis is that the direction of horizontal transfer was in fact from *Aedes *into *Wolbachia*, and AAEL004181 is part of a family of SGS-type genes that originated and evolved in mosquitoes. The acquisition of host genes by *Wolbachia *has not previously been documented. It could be argued that this scenario is more parsimonious since only one inter-domain HGT event would be required, if a subsequent transfer from *w*Pip (or a related strain) to *w*Mel is assumed. In contrast, the hypothesis of *Wolbachia *to *Aedes *transfer requires a different origin of the SGS genes compared to AAEL004181. However, phylogenetic reconstruction (Figure 4) does not support the hypothesis of a single host-to-*Wolbachia *HGT, since AAEL004181 clusters with the *w*Pip gene WP1346 with a posterior probability of 1 and a boostrap value of 89. If AAEL004181 had been transferred from mosquito to *Wolbachia *followed by subsequent transfer between *Wolbachia *strains, the *w*Pip and *w*Mel sequences would be expected to be more closely related to each other than to AAEL004181 and thus cluster together in the phylogenetic tree, which is not the case. Thus, both phylogenetic evidence and the lack of introns support a *Wolbachia*-to-host direction of transfer of AAEL004181. The SGS genes may also have had a bacterial origin, as suggested by their apparent lack of introns, but if this is the case then the HGT event or events responsible would be separate from that involving AAEL004181 (and probably pre-date it, given their greater distance from the *Wolbachia *genes).

## Conclusion

The data presented provide a robust case for HGT between *Wolbachia *and mosquitoes, and we consider *Wolbachia*-to-host to be the most likely direction of this transfer of the genes AAEL004181/8 in *Ae. aegypti *and *mascarensis*. Our results support the argument that HGT between *Wolbachia *and their insect hosts has led to the acquisition of evolutionary innovation, provide a putative mechanism for transfer via nuclear-phage recombination, and suggest that the previously documented examples of recent/ongoing *Wolbachia*-host HGT may have considerably more significance than interesting, but transient, phenomena.

## Methods

Mosquito DNA and RNA extraction, PCR, sequencing, and RT-PCR using a Qiagen one-step RT-PCR kit were carried out as previously described [22,28]. DNA from Museum specimens was extracted using a Qiagen QIAamp DNA micro kit according to the manufacturers instructions. Primers were designed using Primer3 [29] and previously published primers for gene *wsp *(81F and 691R)[30] were used to check for presence of *Wolbachia*, and AegS7F and R primers amplifying the ribosomal *S7 *gene [31] as controls for *Aedes *DNA quality. DNA from *Aedes simpsoni, heischi, soleatus, calceatus, metallicus *and *mascarensis *(all considered phylogenetically close to *Ae. aegypti*) was extracted from preserved Museum specimens; only data from specimens where strong AegS7 amplification was observed were included. DNA from *Ae. aegypti *and *Ae. albopictus *was extracted from laboratory specimens.

### Quantitative RT-PCR

Gene expression levels were monitored using quantitative RT-PCR (qRT-PCR). Total RNA was extracted with TRIzol™ reagent from groups of ten *Aedes aegypti *females and cDNA was synthesized from 1 microgram of total RNA using SuperScript II enzyme (Invitrogen) following the manufacturer's protocol. qRT-PCR was performed on a 1 in 20 dilution of the cDNAs using dsDNA dye SYBR Green I. Reactions were run on a DNA Engine thermocycler (MJ Research) with Chromo4 real-time PCR detection system (Bio-Rad) using the following cycling conditions: 95C for 15 minutes, then 45 cycles of 95C for 10s, 59C for 10s, 72C for 20s, with fluorescence acquisition at the end of each cycle, then a melting curve analysis after the final one. The cycle threshold (Ct) values were determined and background fluorescence was subtracted. Gene expression levels of target genes were calculated, relative to the internal reference gene RpS17 (ribosomal protein S17). Primer pairs used to detect target gene transcripts were as follows: AAEL004181 (forward: 5'-GTT TCC GCA GAA GAA TCA GC-3', reverse: 5'-AGT TCG TCT CCA AAG CAG GA-3'); AAEL004188 (forward: 5'-TGA ATT GCT GCT ACG GTT TG-3', reverse: 5'-TGA ATG GGT CTT TGT GTC CA-3'); Actin5C (forward: 5'-ATC GTA CGA ACT TCC CGA TG-3', reverse: 5'-ACA GAT CCT TTC GGA TGT CG-3') and control RpS17 (forward: 5'-CAG GTC CGT GGT ATC TCC AT-3', reverse: 5'-CAG GAC ATC ATC GAA GTC GA-3').

#### Microarray experiments

Custom *Ae. aegypti *microarrays were designed using Agilent eArray software [32]. A probe set, containing two unique 60-mers per annotated *Ae. aegypti *gene, was designed using the gene expression probe design module. These probes were randomly position across the surface of the array.

*Aedes aegypti *were reared using standard procedures to either 0–2 days (young) or 14–16 days (old) post-eclosion before collection. Total RNA was extracted from pools of 10 female *Ae. aegypti *using Trizol reagent (Invitrogen) and following the manufacturer's protocol. Isolated total RNA was quantified on the Nanodrop ND-1000 spectrophotometer. The two-colour low RNA input linear amplification kit PLUS kit (Agilent Technologies) was used to amplify cyanine-3 (Cy3) and cyanine-5 (Cy5) labeled complimentary RNA (cRNA) from 1.5 μg of total RNA from each pool of female mosquitoes. cRNA samples were purified using RNeasy mini kits (Qiagen), then cRNA concentration and dye incorporation (labeling efficiency) where quantified using the Nanodrop. Prepared cRNA samples were hybridized to four 4 × 44K format microarrays using Agilent reagents and protocols. Microarray slides were washed following Agilent protocols to prevent ozone degradation and scanned with an Agilent scanner at 5 μm scan resolution using the extended dynamic range (XDR) function (XDR Hi 100%, XDR Lo 10%). Agilent feature extraction software (version 9.5.3) was run on all array datasets using the GE2-v5_95_Feb07 protocol. This protocol reports processed Cy3 and Cy5 signal intensities and identifies probes significantly expressed above background (2-sided t-test; P = 0.01).

#### Phylogenetic analysis

The two genes WP1346 and WP1348 in *Wolbachia *strain *w*Pip and the two genes WD0512 and WD0513 from *Wolbachia *strain *w*Mel were concatenated. The gene AAEL004181 from *Aedes aegypti *was extended to the new start codon and the parts annotated as introns were included. All genes identified as putative SGS family members by searching the genomes of *Anopheles gambiae, Aedes aegypti *and *Culex quinquefasciatus *with tblastn in VectorBase [33] were extracted. All gene sequences were, if necessary, extended to the putatively correct start codon based on homology with AAEL004181 and introns were included and translated together with the exon sequences. The gene annotated as CPIJ007816 contained two large open reading frames that were both similar to the SGS genes, they were both included and are called CPIJ007816a and b. Two SGS genes from *Anopheles gambiae*, agSGS2 and agSGS3 sequenced by Korochkina et al. [27], were retrieved separately from Genbank, since the corresponding region in the *Anopheles gambiae *genome contains sequence gaps. The amino acid sequences were aligned using MUSCLE [34]. Phylogenetic reconstruction was performed using MrBayes 3.12 [35], with the mixed amino acid model. The program was run for 200,000 generations, sampling every 100^th ^generation, using 2 runs with 4 chains in each. A consensus tree was constructed using a burnin of 25% of the sampled trees. A maximum likelihood analysis was conducted using RAxML ver. 7.0.4 [36]. A rapid boostrap analysis using 1000 replicates with a following search for the best scoring ML tree was conducted in two separate runs using the WAGF+GAMMA+I model. For each run, the final ML optimization was conducted for every 5^th ^bootstrapped tree to search for the best scoring ML tree.

Sequences are deposited in GenBank accession numbers FM958472–FM958475.

## Authors' contributions

LK and SPS contributed experimental conception/design, genome analysis and manuscript drafting. Phylogenetic analysis was carried out by LK.  ZK and TW performed PCR and RT-PCR experiments.  PC and ZK carried out microarray experiments and analyses.
